# Using molecular dynamics simulations to prioritize and understand AI-generated cell penetrating peptides

**DOI:** 10.1038/s41598-021-90245-z

**Published:** 2021-05-20

**Authors:** Duy Phuoc Tran, Seiichi Tada, Akiko Yumoto, Akio Kitao, Yoshihiro Ito, Takanori Uzawa, Koji Tsuda

**Affiliations:** 1 School of Life Sciences and Technology, Tokyo Institute of Technology, 2-12-1, Ookayama, Meguro-ku, Tokyo 152-8550 Japan; 2 Emergent Bioengineering Materials Research Team, RIKEN Center for Emergent Matter Science, 2-1 Hirosawa, Wako, Saitama 351-0198 Japan; 3 Nano Medical Engineering Laboratory, RIKEN Cluster for Pioneering Research, 2-1 Hirosawa, Wako, Saitama 351-0198 Japan; 4 Graduate School of Frontier Sciences, The University of Tokyo, 5-1-5 Kashiwa-no-ha, Kashiwa, Chiba 277-8561 Japan; 5 RIKEN Center for Advanced Intelligence Project, RIKEN, 1-4-1 Nihombashi, Chuo-ku, Tokyo 103-0027 Japan; 6 Research and Services Division of Materials Data and Integrated System, National Institute for Materials Science, Tsukuba, Ibaraki 305-0047 Japan

**Keywords:** Biological physics, Membrane biophysics, Computational biophysics, Permeation and transport, Computational biology and bioinformatics, Machine learning

## Abstract

Cell-penetrating peptides have important therapeutic applications in drug delivery, but the variety of known cell-penetrating peptides is still limited. With a promise to accelerate peptide development, artificial intelligence (AI) techniques including deep generative models are currently in spotlight. Scientists, however, are often overwhelmed by an excessive number of unannotated sequences generated by AI and find it difficult to obtain insights to prioritize them for experimental validation. To avoid this pitfall, we leverage molecular dynamics (MD) simulations to obtain mechanistic information to prioritize and understand AI-generated peptides. A mechanistic score of permeability is computed from five steered MD simulations starting from different initial structures predicted by homology modelling. To compensate for variability of predicted structures, the score is computed with sample variance penalization so that a peptide with consistent behaviour is highly evaluated. Our computational pipeline involving deep learning, homology modelling, MD simulations and synthesizability assessment generated 24 novel peptide sequences. The top-scoring peptide showed a consistent pattern of conformational change in all simulations regardless of initial structures. As a result of wet-lab-experiments, our peptide showed better permeability and weaker toxicity in comparison to a clinically used peptide, TAT. Our result demonstrates how MD simulations can support de novo peptide design by providing mechanistic information supplementing statistical inference.

## Introduction

Cell penetrating peptides (CPPs) are a family of peptides that can pass though cell and tissue membranes with no interactions with specific receptors^1^. Some effectors such as proteins, small drugs and siRNAs can hardly access intracellular targets due to their hydrophilicity. To enable cellular intake, a CPP is conjugated with an effector by covalent bonds or noncovalent complex formation. Various preclinical and clinical studies using CPPs are being undertaken for diseases such as cerebral ischemia, Alzheimer disease and cancer^1^. In 1988, trans-activator of transcription (TAT) protein of HIV-1 was discovered as the first CPP^2^. Most CPPs reported so far have their origins from natural proteins. Known CPPs such as Penetratin^3^ and Pep-1^4^ are classified into categories such as cationic, amphipathic and hydrophobic due to sequential features. To date, there are no clear rules to distinguish if a peptide is cell-penetrating or not. Also, we need to make sure that CPPs are non-toxic for safety in therapeutic applications.

In this paper, we build a computational pipeline for automated discovery of CPPs to expand the existing toolbox of CPPs and contribute to future applications. Recently, deep-learning-based peptide generators are increasing popularity^5–8^. Given a training set of functional peptides, the generator can create similar peptides that shares common statistical features with known peptides. For example, Tallorin et al.^6^ discovered novel peptide substrates for enzymes using machine learning. Tucs et al.^5^ and Nagarajan et al.^8^ generated antimicrobial peptides and validated their activity in wet-lab experiments. Due to active research about deep learning methods, the generators are being improved towards higher throughput and better statistical fidelity. A less discussed yet crucial problem in an automated discovery pipeline is in the prioritization process by a human expert^9^. Due to resource limitation, the number of peptides that can be verified by wet-lab experiments is limited. To avoid wasteful experiments, an expert has to review all generated sequences and choose a few of them for validation. For CPPs, it is an extremely difficult task, because biomembrane penetration is a dynamic process and it is very difficult to predict dynamic behaviour of the peptide from a sequence alone.

In our pipeline, peptide sequences are prioritized via a mechanistic score obtained by analysing the trajectories of molecular dynamics (MD) simulations. To this aim, a membrane model is constructed and placed in the x–y plane and the peptide in question is placed above the membrane. After MD simulation, some peptides get through the membrane showing permeability, while others cannot. To quantify membrane permeability, the free energy difference before and after penetration would be an ideal measure. Due to prohibitive computational cost of free energy calculation, however, we employ the following steered MD simulation, which has been successfully applied to protein–ligand dissociation^10^. A virtual spring which connects a dummy atom and the peptide is considered. The dummy atom moves into the cell membrane in constant velocity, followed by the peptide connected to the spring. When the peptide has difficulty in moving forward, the spring is elongated and the applied force by the spring (i.e., steering force) is increased. We take the maximum steering force during the whole penetration process as our permeability measure.

When dealing with novel sequences, their three-dimensional structures are unknown, but initial structures are necessary for starting MD simulations. In this paper, the structures are predicted by a homology modelling software called I-TASSER^11^. It turned out that the predicted structures are quite variable, and it is highly possible that the trajectory obtained by a simulation is not reliable. To ensure that the trajectory is close to reality, simulations with multiple initial structures should at least show a consistent movement pattern, resulting in stable values of steering force. To select peptides with low and stable steering forces, we employ a recently proposed machine learning framework called sample variance penalization^12^. In machine learning, model parameters are determined by minimizing loss average over all examples. Maurer and Pontil found that minimizing loss variance is also beneficial to achieve best prediction accuracy^12^. To this aim, they proposed to minimize the sum of loss average and a penalization term to keep the variance low. Based on the same idea, the mechanistic score is defined as the sum of steering force average and the penalization term.

The mechanistic score is calculated for all generated sequences, and the top-scoring peptide called Pep-MD was selected for detailed analysis. Pep-MD showed consistent conformation change in all simulations: membrane entry is guided by hydrophobic residues. Inside the membrane, charged residues swap interaction partners from top lipid headgroups to bottom headgroups to open the way out of the membrane. Additional MD simulations are performed for 100 shuffled sequences and their steering forces are measured. As a result, the steering force of Pep-MD was shown to be exceptionally low in comparison to the shuffled sequences. To investigate essential residues of Pep-MD, the consensus sequence of Pep-MD and low-steering-force shuffled sequences is obtained via multiple alignment. As a result, we found that the positions of charged residues in Pep-MD are essential in determining cell permeability.

Encouraged by the promising results in dynamics analysis, Pep-MD went on to in vitro validation. First, a cell viability assay found that Pep-MD is less toxic than widely-used TAT. Using confocal laser scanning microscopy, it is confirmed that Pep-MD distributes inside the cell membrane. Quantitative analysis using fluorescence activated cell sorting (FACS) showed that Pep-MD has better permeability than TAT. This result shows that, despite uncertainty in predicted peptide structures, MD simulations can be made useful for selecting promising sequences from AI-generated unannotated sequences, and can provide useful information for decision making by scientists.

## Results

### Sequence generation

Cell penetrating peptides of 20 amino acid in length are generated with machine learning. As training examples, we collect sequences not longer than 52 amino acids from the following databases: APD^13^, CAMP^14^, LAMP^15^, DBAASP^16^. Sequences with non-natural amino acids are removed to avoid difficulties in force-field parameterization. Redundant sequences are removed via multiple sequence alignment with cut-off ratio 0.35. The final dataset contains 16,648 sequences of positive data (cell penetrating) and 5583 sequences of negative data (non-penetrating).

We use a recurrent neural network (RNN) consisting of two serially connected gated recurrent units (GRUs)^17^, each with 256 hidden variables. It is trained with the positive data only. A sequence is generated by starting from a random prefix and elongating it up to length 20 with the RNN. We created 10^7^ sequences from the same number of prefixes whose lengths are randomly chosen in the range from 1 to 20. To reduce the number of generated sequences, another neural network called permeability predictor is employed. Given a sequence, it computes a 256-dimensional hidden vector at each position using a GRU. The hidden vector is fed to a one-layer dense neural network to yield a partial score at each position. Finally, it is summarized to a final likelihood score by max-pooling. The likelihood score is normalized from 0 to 1 and a higher value indicates that the sequence is more likely to belong to the positive class. All the generated sequences are processed by the permeability predictor, and top 100 sequences according to the likelihood score are selected. Next, the set of sequences is further reduced by the synthesizability score obtained from a web service provided by Thermo Fisher^18^. Twenty-four peptides with synthesizability higher than or equal to three were selected for the next step.

All the neural networks were implemented using Keras package^19^ and trained by the Adam algorithm^20^. The networks were trained up to 100,000 epochs with careful monitoring of loss and accuracy until convergence.

### Sequence prioritization by MD simulations

For each peptide with high likelihood and sufficient synthesizability, five initial structures are prepared by I-TASSER^11^. We use default setting of I-TASSER including the non-redundant database, BLASTP sequence alignment, REMC simulations, re-assembly of models, and ranking stages. For cell penetration simulation, a membrane model involving 200 POPC (1-palmitoyl-2-oleoyl-glycero-3-phosphocholine) lipids and 50 cholesterol molecules was prepared. The membrane is placed at the x–y plane and an initial peptide structure is placed above it. In steered MD simulation, the center of mass of the peptide is directly connected to a head of a virtual spring, while the other head is connected to a dummy atom moving into the membrane with constant velocity 1 nm ns^−1^. The force constant of the spring is 1000 kJ mol^−1^ nm^−1^. The steered molecular dynamics simulation is performed up to 50 ns. The spring length changes over time, and we recorded the maximum length and corresponding steering force, i.e., the force applied by the spring to the peptide. Low steering force implies that the peptide has high permeability.

Due to variability of initial structures and stochasticity of the barrier crossing event, the steering force has a certain level of diversity, where the mean and standard deviation are reported in Table 1. Using sample variance penalization^12^, the mechanistic score is computed as $m+\lambda \sqrt{v/n}$ where *m* is the mean, *v* is the variance, $n$ is the number of samples and $\lambda$ is a trade-off parameter. Here, $\lambda$ is set to 2.5 according to Maurer and Pontil^12^. We ranked the peptides according to the mechanistic score and selected the top-ranked peptide YRGWHCRGITKNGIIFDIKW (Pep-MD) for synthesis and validation.

**Table 1:** Top 10 peptides ranked according to the mechanistic score.

Peptide	Mean	Standard deviation	Mechanistic score
YRGWHCRGITKNGIIFDIKW	621.3	6.0	628.0
DIGWDTEHPPKTCQIICSVI	648.6	26.5	678.2
KIGHQKIIGETQKRCFFQWV	639.1	36.7	680.1
LMQPVTKICTNGHCTGQFFG	681.5	9.7	692.3
RTCKRVHLGVLNVFNTCHYC	643.5	69.5	721.2
IGGVKAIMSRERPIKIKCTK	668.9	55.0	730.4
ELMLNGWISAGTCQRIWGSR	615.1	117.6	746.6
PCIKIFFCKSLFCRNVRETK	766.7	20.7	789.9
FRHCFPPQAQCTVEIGWVDI	699.2	89.4	799.1
FIWGKVAIRCFHNTFCKRHC	645.5	146.5	809.2

The mean and standard deviation of the steering force (kJ mol^−1^ nm^−1^) in five steered MD simulations are reported. These two values are summarized into the final mechanistic score via sample variance penalization.

### Dynamics of membrane penetration by the peptide

**Figure 1:**
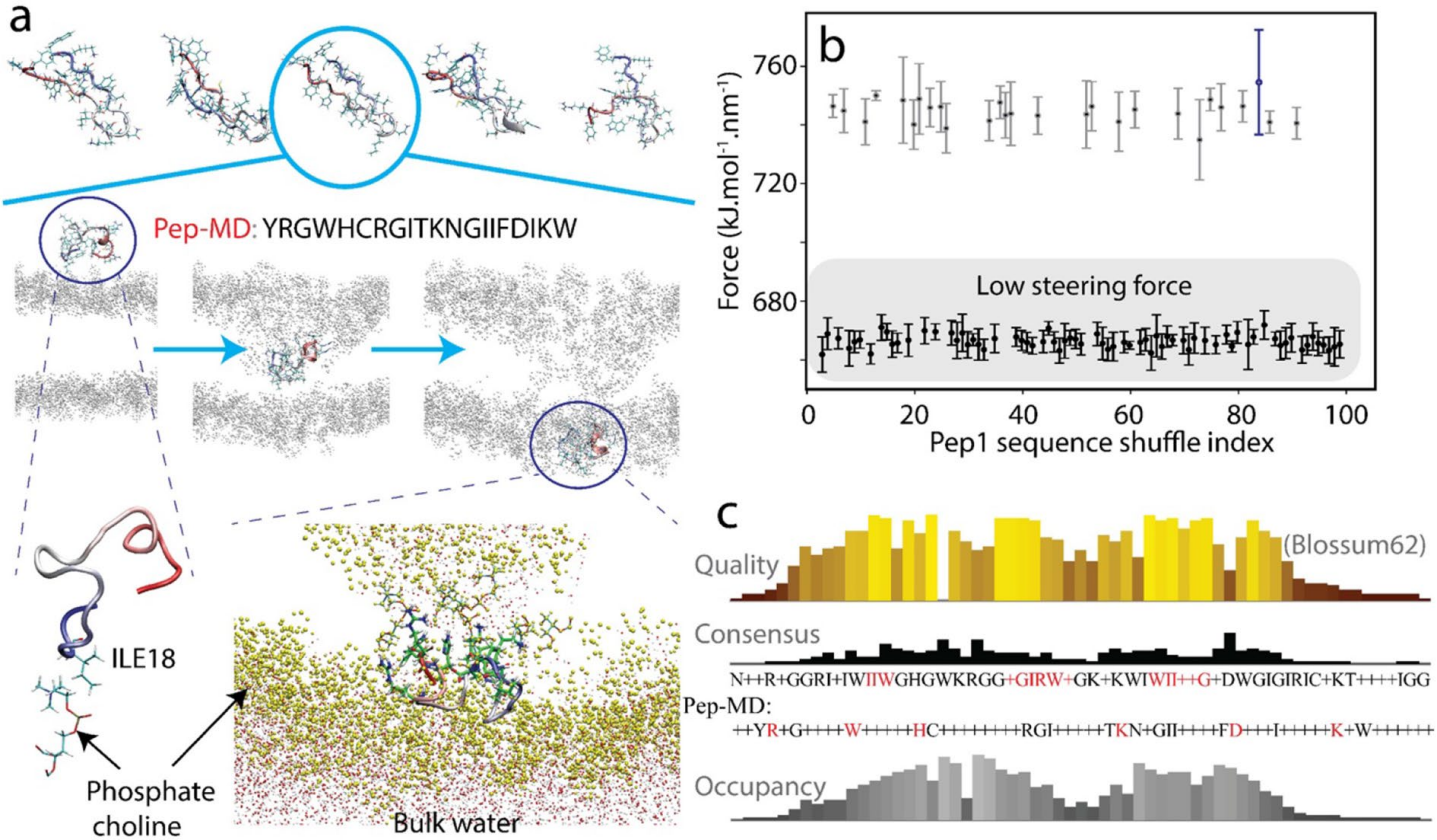
Atomic mechanism of the penetrating process of Pep-MD. (**a**) Snapshots from the penetration process. The top row: the five conformations of the peptides predicted by I-TASSER. The middle row: the penetration process. The gray dots illustrate lipid headgroups. The bottom row: the left image shows a closeup view of Pep-MD and a phosphate choline at the first contact; the right image shows the stage just before the escape from the membrane. Yellow dots represent the membrane headgroups while red (oxygen) and white (hydrogen) spheres represent water molecules. (**b**) Distribution of the maximum steering force of shuffled peptides. The blue dot and error bar indicate the largest steering force among them. (**c**) The results of the sequence alignment (quality, consensus and occupancy) among the peptides with low steering force by Blossum62. The sequence shown with the consensus indicates the alignment motif (the upper sequence), whose high consensus probability/occupancy residues are shown in red. The sequence of Pep-MD (the lower) is also shown; the residues shown in red match the consensus sequence.

We investigated the mechanisms of the membrane penetration by looking into the inter-atomic interactions between the peptide and membrane during the five MD simulations. Pep-MD contains seven hydrophobic, five positively-charged, one negatively-charged residues. The simulated membrane is composed of cholesterols and POPC lipids, the latter containing the amphiphilic headgroup (phosphatidylcholine) with a positively-charged choline group and a negatively-charged phosphatidyl group. In all trials, Pep-MD made first contacts with the membrane by inserting its hydrophobic residues (Tryptophan in 3 cases) and (Isoleucine in 2 cases) as shown in Movies S1–S5 and Fig. 1a. After that, the initial contacts help Pep-MD to embed itself into the hydrophobic region of the membrane, while the charged residues tend to keep interactions with the lipid headgroups. The insertion also caused the bending or thinning of the membrane. Subsequently, the polar and charged residues of the peptides swapped the interacting partners to the head groups of the other side, opening the exit pathway to complete the penetration.

To examine conformational change of the peptide during this process, we performed the detailed analysis of the structural change, hydrogen bond and hydrophobic contacts (Figs. S1 and S2). The average number of intra-Pep-MD hydrogen bonds with the membrane was 11.6 at the first contacts. After that, we observed slight increase of the number in two cases while it decreased in the other 3 cases (top panels in Fig. S1), which does not show no clear tendency in the change of hydrogen bonds during the penetration. Although the lipid head groups are situated in both sides of the membrane, the number of hydrogen bonds between Pep-MD and lipid headgroups (9.4 bonds on average) did not exhibit a binary-peak tendency as a function of time, and rather shows a single peak (the panels of the 2nd row in Fig. S1). This is consistent with the fact that the peptide swapped the hydrogen bonding partners from the one side to the other. This single-peak feature is also seen in the number of Pep-MD/lipid tail contacts (the panels of the 3rd row in Fig. S1. 279.6 contacts at the peak). During the penetration, intra-peptide hydrophobic contacts did not largely change but the total number of hydrophobic contacts became significantly high (the bottom panels in Fig. S1), which probably enables the peptide to gain hydrophobic interactions, driving the penetration.

We also examined the time evolution of the secondary structure during penetration (left of Fig. S2). As shown in the top of Fig. 1a, the five initial structures before the penetration are significantly different. In the 1st, 3rd and 5th trials where the middle regions of the peptide tended to form a 3_10_, α or π helix, the structure did not drastically change during the penetration. In contrast, in the 2nd and 4th trials, significant change was seen in the last step when the peptide escaped into solvent. This is also consistent with the time evolution of the root-mean-square deviation (RMSD) from the initial structure (right of Fig. S2) in which drastic increase of RMSD was observed at the time of the escape.

To evaluate the dependency of sequential position of each amino acid in Pep-MD in cell permeability, we randomly permutated the peptide sequence, keeping the amino acid components. The structures of the 100 shuffled sequences were then modelled with I-TASSER and their membrane penetration process was simulated with the same method as in the case of Pep-MD. We showed maximum values of the steering force and their standard deviation in Fig. 1b. The maximum steering forces split into two distinctive groups, high (> 720 kJ mol^−1^ nm^−1^) and low (< 680 kJ mol^−1^ nm^−1^) groups. It is also worth mentioning that all these values were significantly higher than that of Pep-MD (621.3 ± 6.0 kJ mol^−1^ nm^−1^). Among the high group, the peptide with the sequence TRDRCGIGIWIGKYNWFHKI yielded the highest maximum steering force (blue in Fig. 1b). This peptide forms a complete coiled structure, consistent with the fact that the peptides with higher helical propensity tended to have more ability to penetrate. Next, to identify the sequence motifs that contribute to lower the maximum steering force, we performed multiple sequence alignment among the low-force group indicated in Fig. 1c. We found three motifs having high occupancy and consensus, i.e., IIW, -GIRW- and WII-G (shown in red). The second and third motifs show some similarity with the sequence of Pep-MD. Interestingly, the positions of the several charged residues (R2, H5, R7, K11, D17, and K19) in Pep-MD are identical with or very close to those in the consensus sequence, implying that the positions of charged residues are important for cell permeability.

### Validation by experiments

**Figure 2:**
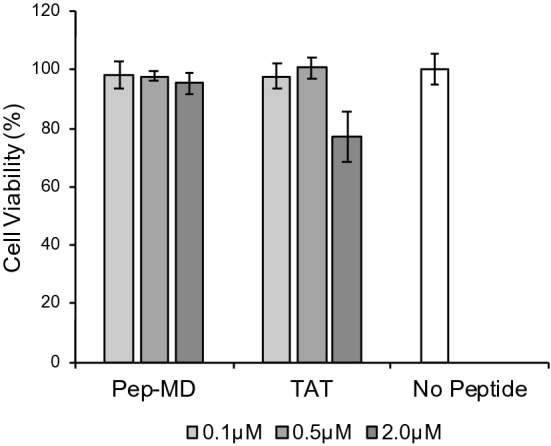
Cell viability test clearly indicated that Pep-MD has little toxicity at even as high as 2.0 µM, although TAT exhibited cell toxicity at 2.0 µM. Errors represent standard deviation (n = 3).

We first evaluated cell toxicity of Pep-MD. Specifically, we quantified HeLa cell viability using CellTiter-Glo® Luminescent Cell Viability Assay kit in the presence of a series of concentration of Pep-MD, 0.1, 0.5 and 2 µM, in serum-free cell culture media. The kit can visualize the number of viable cells as chemiluminescence intensity based on quantitation of the ATP present, an indicator of metabolically active cells. As a positive control, we also evaluated TAT (GRKKRRQRRR in amino acid sequence). Figure 2 shows that Pep-MD has low toxicity at all concentration levels. In contrast with Pep-MD, TAT exhibited significant cell toxicity at 2.0 µM, indicating that Pep-MD is less toxic than TAT.

**Figure 3:**
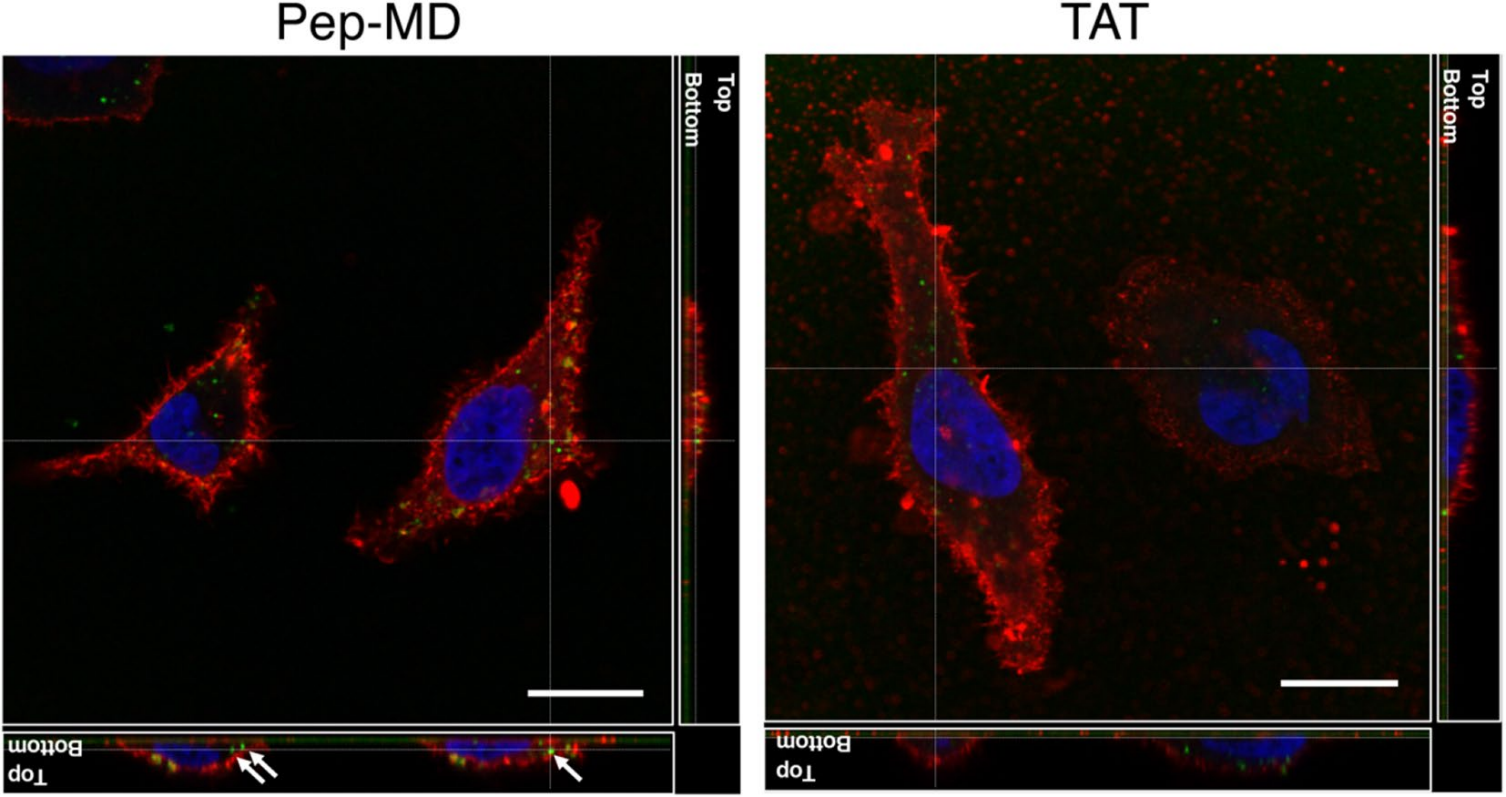
Intracellular distribution of Pep-MD and TAT at single-cell resolution using confocal laser scanning microscopy (left and right, respectively). The arrows in the left figure indicate green fluorescence spots corresponding to Pep-MD inside the cell membrane (red fluorescence), clearly indicating that Pep-MD is cell permeable.

Next, we experimentally validated the cell internalization of Pep-MD at single-cell resolution using confocal laser scanning microscopy. To visualize Pep-MD in cells, we synthesized the fluorescein-modified Pep-MD and TAT. Fluorescein is a well-used green fluorescence dye in microscopy. After HeLa cells are incubated in the presence of 0.1 µM of each peptide (Pep-MD and TAT) for 4 h, we washed away the peptides with phosphate buffered saline and observed the intracellular distribution of green fluorescence from Pep-MD or TAT in HeLa cells. As shown in Fig. 3, we found green fluorescence from Pep-MD inside the cell membrane, clearly indicating that Pep-MD is cell permeable.

**Figure 4:**
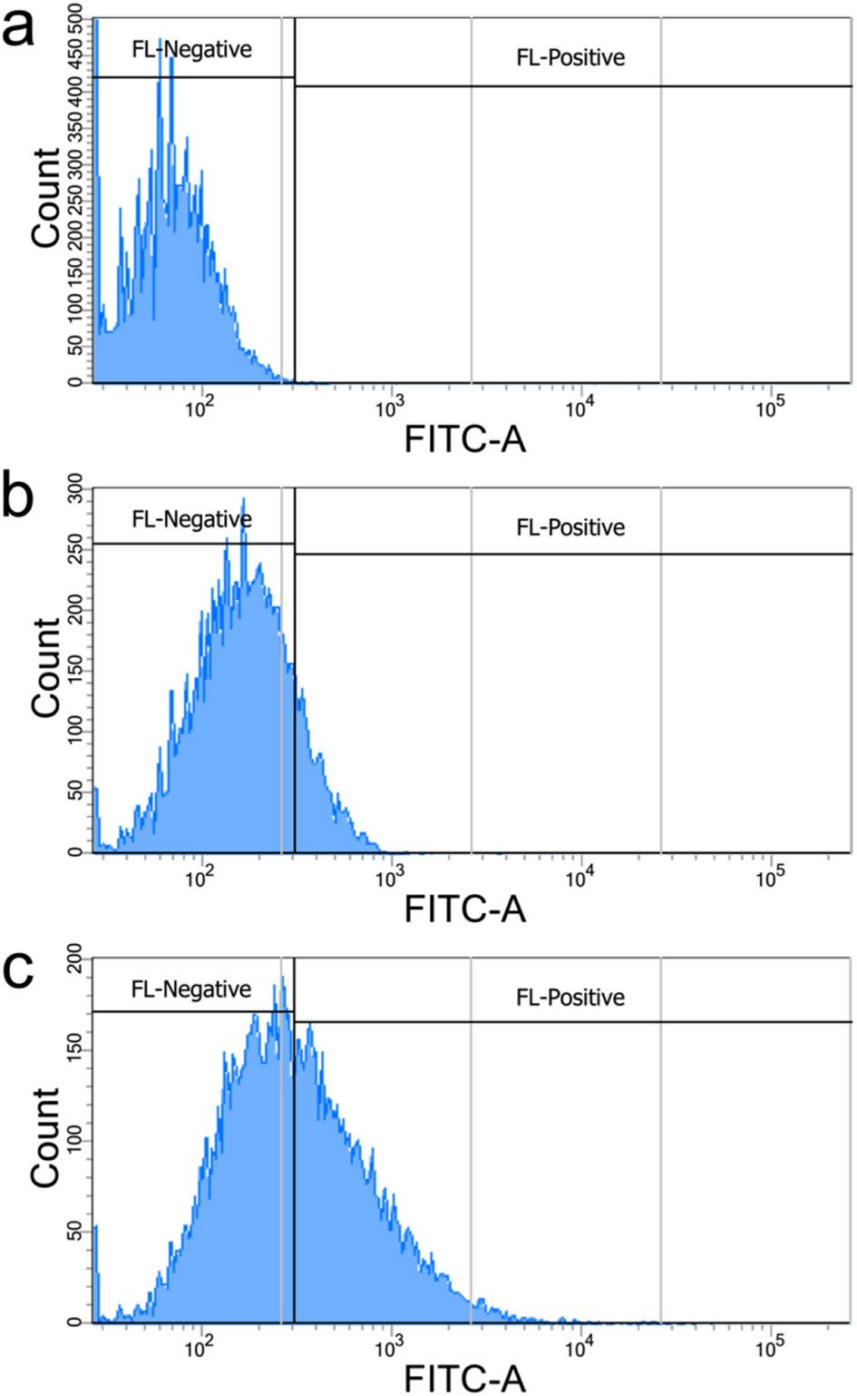
Effective cell-internalization of Pep-MD confirmed by FACS. (**a**) Distribution of fluorescence intensity in the control Hela cells in absence of any peptide. A threshold discriminating fluorescence (FL) negative and positive cells is defined at the tail of the distribution. (**b**) Intensity distribution of the cells incubated with TAT. Only 14% of the cells are FL-positive. (**c**) Intensity distribution with Pep-MD. More than 45% of the cells are FL-positive.

To address the efficiency of Pep-MD cell-internalization, we analysed the Pep-MD-internalized HeLa cells using fluorescence activated cell sorting (FACS). FACS can quantify the green fluorescence intensity for each cell. The HeLa cells which were incubated in the presence of 0.1 µM peptides (Pep-MD or TAT) for 4 h were subjected to FACS. As shown in Fig. 4, we found that more than 45% of cells which were incubated in presence of Pep-MD exhibited higher fluorescence than the threshold defined by the control cells cultured with no peptides. In contrast, only 14% of cells with TAT exhibited higher fluorescence than the threshold. This clear difference indicates that Pep-MD is more cell-permeable than TAT.

## Discussion

MD simulations have been used to analyse mechanisms of experimentally observed phenomena and barely used to discover new molecules. We presented a computational pipeline involving MD simulation at the core and successfully found a new CPP called Pep-MD. Here, MD simulations are used for two purposes: one is to calculate the mechanistic score that ranks machine-learning-generated sequences. The other is to understand the peptide by analysing its dynamic behaviour. This analysis is supposed to help scientists make a decision to perform expensive in vitro experiments. The membrane composition in our MD simulations was adjusted to that of typical human cells. The dependence of cell permeability to membrane composition is not clear at this point, but it would be an interesting topic for further studies.

Most studies about machine-learning-based generation focus on efficiency and accuracy of machine learning methods, ignoring human aspects^7^. In a real development pipeline, scientists are inevitably involved and supporting them to make right decisions would be more important than the performance of machine learning methods^9^. Our case study showed how a successful pipeline is made with MD simulations. In future, our pipeline may serve as a template for AI-based development projects of not only peptides, but also proteins^21^, chemical compound^22^ and inorganic materials^23^.

## Methods

### Membrane model

The membrane model is aligned into x–y plane, solvated in rectangular box 9.2589 × 8.9667 × 11.8224 nm with TIP3P water model keeping ionic concentration at 0.15 M. The model is first energy-minimized by using the steepest descent algorithm with 1000 kJ mol^−1^ nm^−1^ positional restraint of all heavy atoms. The model is then heated from 0 to 100 K using NVT ensemble in 0.5 ns, then switching to anisotropic NPT ensemble to keep the pressure at 1.0 atm, increasing the temperature from 100 to 300 K within 0.5 ns with 1000 kJ mol^−1^ nm^−1^ positional restraint of all heavy atoms. The model is then thermalized in NPT ensemble at 1.0 atm pressure and 300 K in another 1 ns, which the deduction of 100 kJ mol^−1^ nm^−1^ in positional restraint force constant at every 0.1 ns. The membrane model is then relaxed without positional restraints in NPT ensemble at 1.0 atm pressure and 300 K in another 100 ns. In this stage, all the simulations were carried out in AMBER 18 package by using Langevin thermostat^24^ and Berendsen barostat^25^ to mimic the simulation condition. The last snapshot in this stage is then extracted and converted to a GROMACS compatible format for the latter simulations.

### Inserting the peptide

The peptide structure predicted by I-TASSER is inserted in the solvent part of the simulation box to align the center of mass of the peptide to the center of mass of the box, keeping the minimum distance with the membrane at least 1.0 nm. The overlapping water molecules and ions with peptides are removed. Ions is then added to keep the charge neutrality and ionic concentration at 0.15 M. The system is energy-minimized by using the steepest descent algorithm with 1000 kJ mol^−1^ nm^−1^ positional restraint of all heavy atoms of peptide and all-atoms of lipid membrane, and heating up from 0 K up to 300 K in 1.0 ns with NVT ensemble. We used velocity rescaling^26^ as the thermostat in this stage. The system is then thermalized with NPT ensemble to match pressure at 1.0 atm and temperature at 300 K using velocity rescaling thermostat^16^ and Berendsen barostat^25^ in 10 ns with 1000 kJ mol^−1^ nm^−1^ positional restraint of all atoms of lipid membrane, and C_α_ atoms of the peptide.

### Peptide synthesis

Pep-MD and TAT were synthesized using automated microwave peptide synthesizer (Liberty Blue, CEM, Japan) by following manufacturer’s protocol. Briefly, reagents include; 0.05 mmol resin (Fmoc Rink Amide ProTide Resin, CEM), N,N-dimethylformamide (DMF) as the main solvent, 20% piperidine in DMF for deprotection, 0.2 M Fmoc-amino acids in DMF, 0.5 M N,N′-Diisopropylcarbodiimide (DIC) in DMF as the activator and 0.5 M Oxyma in DMF as the base. Fmoc-amino acids and Oxyma were purchased from CEM and DMF, piperidine, DIC were purchased from Fujifilm Wako Chemicals (Japan). To modify Fluorescein (FAM) to the N-terminus of the synthesized peptides, the peptides on the resins were mixed with 0.02 mM NHS-activated FAM (Thermo fisher scientific, USA) and 0.5 M Oxyma in DMF. After incubation at 50 °C for 1 h, the resins were washed with methanol and the resins were collected. After cleavage the peptides from the resins, peptides were further purified using a high-performance liquid chromatograph (Extrema, JASCO, Japan) equipped with a C18 column (COSMOSIL C18-AR-II, Nacalai Tesque, Japan). A mixture of solvent A (0.1% TFA in water) and B (0.1% TFA in acetonitrile) was used as the mobile phase; and a linear gradient from 5 to 60% B for 60 min at a flow rate of 4.0 mL min^−1^ was applied. The mass of the purified peptides were verified by MALDI-TOF–MS (microflex LT, Bruker daltonics, USA); FAM-TAT, m/z = 1917.360 [M + H]^+^, calc. m/z = 1917.974 (monoisotopic mass for all). FAM-Pep-MD (Fluorescein-YRGWHCRGITKNGIIFDIKW) were synthesized and purified by the support unit for peptide synthesis in RIKEN CBS; FAM-peptide1, m/z = 2821.4 [M + H]^+^, calc. m/z = 2821.3. The peptide was dissolved in ultra-pure water and the concentration of the peptide was determined using the extinction coefficient of FAM (495 nm, 75,000 M^−1^ cm^−1^).

### Cell culture

The HeLa cell line was purchased from RIKEN cell bank (Tsukuba, Japan). HeLa cells were cultured in Dulbecco’s Eagle’s medium (DMEM, FUJIFILM Wako Pure Chemical, Japan) supplemented with inactivated 10% fetal bovine serum (FBS, Biosera, France) and 1% penicillin–streptomycin solution (FUJIFILM Wako Pure Chemical).

### Cell toxicity test

HeLa cells were seeded at a density of 8000 cells per well in 96 well plate (AGC Techno Glass, Shizuoka, Japan). After 24 h incubation, cell culture medium was exchanged with fresh DMEM containing each peptide and 1% (v/v) dimethyl sulfoxide (DMSO) without FBS and cells were cultured for another 24 h. Cell viability of each well was quantified with CellTiter-Glo® Luminescent Cell Viability Assay kit (Promega, Madison, WI, USA) and EnSpire™ microplate reader (PerkinElmer, Waltham, MA, USA) according to manufacturer’s instructions. The cell viability was calculated as the chemiluminescence intensity ratio of samples to that of positive control without peptide.

### Observation of intracellular distribution of peptide

HeLa cells were seeded (at a density of 1.5 × 10^5^ cells/dish) in 35 mm glass bottom dish (AGC Techno Glass) and incubated for 24 h. After cell condition became stable, the cells were incubated with 0.1 µM of each peptide and 1% DMSO at 37 °C for 4 h. The cells were washed with phosphate buffered saline (PBS, FUJIFILM Wako Pure Chemical) three times and cell membrane was stained with 5 µM of Vybrant™ CM-DiI Cell-Labeling Solution (Thermo Fischer Scientific) for 5 min. The cells were washed with PBS and fixed with 4% paraformaldehyde solution (FUJIFILM Wako Pure Chemical) for 10 min. After washing with PBS three times, the cells were mounted with Prolong Diamond Antifade mountant with DAPI (Thermo Fischer Scientific). Intracellular distribution of green fluorescence from the peptide was observed using Leica TCS SP8 confocal laser scanning microscope (Leica Microsystems GmbH, Wetzlar, Germany) at the Support Unit for Bio-Material Analysis in RIKEN Center for Brain Science, Research Resources Division. The fluorescence intensity was adjusted with Leica LAS X software to compare the fluorescence distribution from peptides and cell membrane.

### FACS analysis

HeLa cells were seeded (at a density of 2.2 × 10^5^ cells/dish) in 35 mm polystyrene dish (AGC Techno Glass) and incubated for 24 h. After cell condition became stable, the cells were incubated with 0.1 µM of each peptide and 1% DMSO at 37 °C for 4 h. The cells were washed with PBS three times and collected with 0.25 w/v% trypsin-1 mM EDTA solution (FUJIFILM Wako Pure Chemical). After washing with PBS, the collected cells were fixed with 4% paraformaldehyde solution (FUJIFILM Wako Pure Chemical) for 10 min. The fixed cells were washed with PBS three times and subjected to FACS analysis using FACS Aria III (Becton Dickinson, Franklin Lakes, NJ, USA).

## Supplementary Information

Supplementary Information.

## Data Availability

The code and datasets are available at https://github.com/tsudalab/bopp.
