# Supplementary material for: Using molecular dynamics simulations to prioritize and understand AI-generated cell penetrating peptides
Source: Sci Rep. 2021 May 20;11:10630. doi: 10.1038/s41598-021-90245-z (PMC8137933; doi:10.1038/s41598-021-90245-z)
Supplement: Supplementary file 1 — Supplementary Information. [file 41598_2021_90245_MOESM1_ESM.pdf]

Supplementary information for:

Using Molecular Dynamics Simulations to Prioritize and Understand AI-generated Cell-penetrating Peptides

Duy Phuoc Tran[1], Seiichi Tada[2], Akiko Yumoto[2], Akio Kitao[1], Yoshihiro Ito[2,3], Takanori Uzawa[2,3], Koji Tsuda[4,5,6*]

[1] School of Life Sciences and Technology, Tokyo Institute of Technology, 2-12-1, Ookayama, Meguro-ku, Tokyo 152-8550, Japan.[2] Emergent Bioengineering Materials Research Team, RIKEN Center for Emergent Matter Science, 2-1 Hirosawa, Wako, Saitama 351-0198, Japan. [3]Nano Medical Engineering Laboratory, RIKEN Cluster for Pioneering Research, 2-1 Hirosawa, Wako, Saitama 351-0198, Japan. [4] Graduate School of Frontier Sciences, The University of Tokyo, 5-1-5 Kashiwa-no-ha, Kashiwa, Chiba 277-8561, Japan.[5]RIKEN Center for Advanced Intelligence Project, RIKEN, 1-4-1 Nihombashi, Chuo-ku, Tokyo, 103-0027 Japan.[6]Research and Services Division of Materials Data and Integrated System, National Institute for Materials Science, Tsukuba, Ibaraki 305-0047, Japan.

*e-mail: tsuda@k.u-tokyo.ac.jp

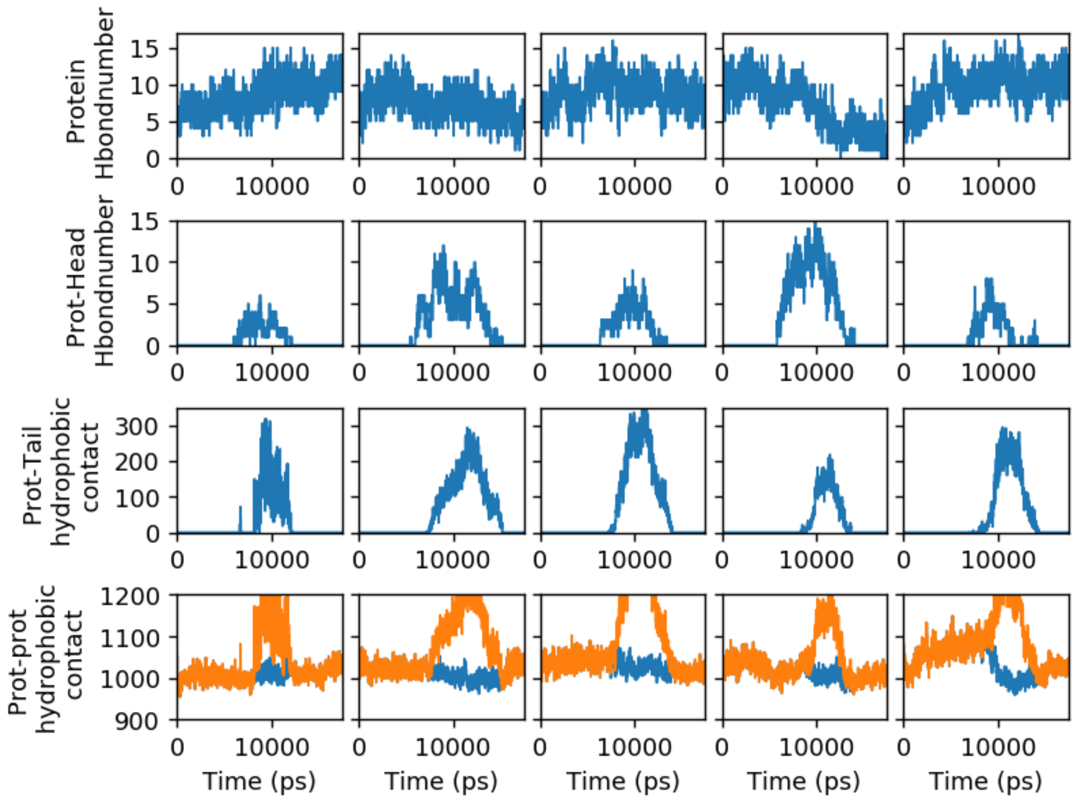

Fig S1. The number of hydrogen bonds within Pep-MD (top five panels) and between Pep-MD and lipid head groups (2nd), the number of contacts between Pep-MD and lipid tails (3rd), and hydrophobic contact within Pep-MD (bottom panels) during the five trials of the membrane penetration of Pep-MD. The orange lines in the bottom panel indicate the sum of hydrophobic contacts within Pep-MD and between Pep-MD/lipid tails.

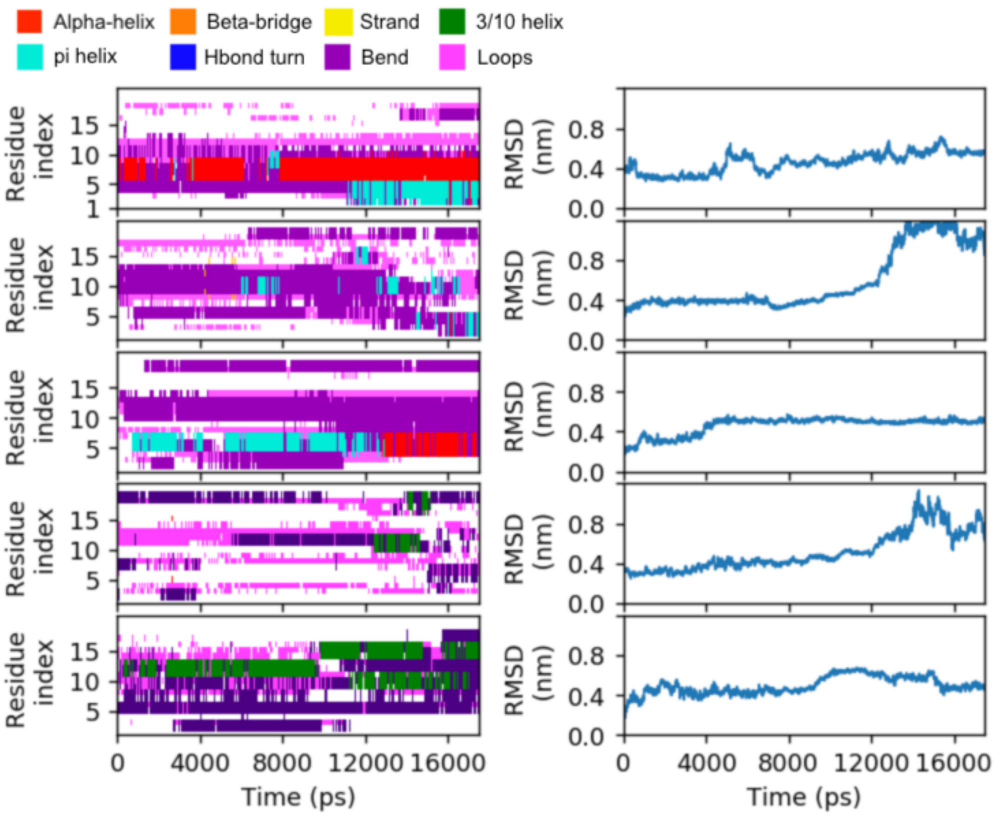

Fig S2. The secondary structure change during the five penetration processes (left) and the root-mean-squared-deviation (RMSD) of the Pep-MD backbone from the initial conformation of each simulation (right).

Movie S1-S5. Penetration process of Pep-MD during the five MD simulations. POPC of the upper and lower leaflets are shown in yellow and green, respectively. Cholesterols are shown in cyan. They can be accessed from the following URL:

S1: https://www.dropbox.com/s/3tkmzs499sf46oa/MovieS1.mp4?dl=0
S2: https://www.dropbox.com/s/fnvc2z0nwcv7k7o/MovieS2.mp4?dl=0
S3: https://www.dropbox.com/s/joc0vk2qyq3qxnf/MovieS3.mp4?dl=0
S4: https://www.dropbox.com/s/blqmosvbyamtg86/MovieS4.mp4?dl=0
S5: https://www.dropbox.com/s/s51vr5epkefmz4m/MovieS5.mp4?dl=0
